# Thermal potentiation improves IFN-γ production but not cytotoxicity in human CAR-T cells

**DOI:** 10.1186/s13104-025-07249-5

**Published:** 2025-04-23

**Authors:** Niladri Bhusan Pati, Yixin Jin, Suman Kumar, Jon Amund Kyte, Rafal Ciosk

**Affiliations:** 1 Department of Biosciences, University of Oslo, Oslo, Norway; 2 Department of Cancer Immunology, Institute for Cancer Research, Oslo University Hospital, Oslo, Norway; 3 Department of Clinical Cancer Research, Oslo University Hospital, Oslo, Norway; 4 Faculty of Health Sciences, Oslo Metropolitan University, Oslo, Norway; 5 Manipal Centre for Biotherapeutics Research, Manipal Academy of Higher Education, Manipal, Karnataka India

**Keywords:** CAR-T, STEAP1, CD8 + T cells, IFN-γ, Cancer immunotherapy, Hyperthermia, Febrile temperature, Thermal medicine

## Abstract

**Objective:**

Body temperature plays an important role in cancer, with febrile temperature generally associated with improved cancer resistance. In murine models, this resistance has been linked to the cytotoxic T cells, whose differentiation into cancer-killing effector cells is poor at lower but robust at elevated temperatures. If conserved, temperature-mediated potentiation of patient-derived T cells could be implemented to improve the existing cancer treatments, including the chimeric antigen receptor T-cell therapy (CAR T-cell therapy). Here, we tested this possibility using human STEAP1 CAR-T cells developed to target prostate cancer.

**Results:**

In mice, transient exposure to febrile temperature (39–40 ºC) increases the production of IFN-γ and the cancer-killing ability of CD8 + T cells. Using a similar temperature treatment, we observed elevated levels of IFN-γ also in the human CAR-T cells. However, these cells displayed no improvement in their ability to kill cancer cells. Although we cannot discount the possibility that alternative protocols might lead to other outcomes, our findings suggest that incorporating thermal potentiation into existing protocols of CAR-T cell therapy may be more complicated than anticipated.

## Introduction

There is increasing awareness that body temperature plays an important role in cancer, with hyperthermia generally inhibiting tumor formation, growth, and metastasis [1]. Exploiting the temperature-sensitivity of cancer cells, hyperthermia is sometimes used as adjuvant in radiation and chemotherapy [2]. The connection between cancer and body temperature has also been studied in animal models, where cancer is more aggressive at sub-thermoneutral temperatures [3, 4]. In mice, these temperature-dependent differences in cancer resistance have been linked to the cytotoxic CD8 + T cells, which are critical components of anti-tumor immune defense [3]. Specifically, the differentiation of naive CD8 + T cells into cancer-killing effector cells is worse at lower (33 ºC) but better at higher temperatures (39.5–40 ºC) [5]. This holds true both in vivo and in vitro, as isolated CD8 + T cells transiently exposed to febrile temperature (39-39.5 ºC) and introduced into a host are more effective combating cancer [6]. In this study, we refer to the in vitro stimulation of CD8 + T cells by febrile temperature as the *t*hermal *p*otentiation of *T* cells (TPT).

Cell therapy employing tumor targeting chimeric antigen receptors (CARs) is approved and effective against leukemia, lymphoma, and myeloma, even when given to heavily pretreated patients [7, 8]. In this therapy, CD8 + T cells obtained from the patient are expanded in the laboratory, modified to make them more effective in killing cancer cells, and then delivered back to the patient to help the immune system fight cancer [9–11]. Since the ex vivo expansion of CAR T cells is potentially compatible with TPT, we tested if transiently exposing CAR T cells to febrile temperature may improve their cancer-killing ability. As TPT has been demonstrated so far only in mouse, we tested the effect of febrile temperature on human CAR-T cells. Specifically, we used CAR-T cells that we have developed for targeting the protein STEAP1 (six-transmembrane epithelial antigen of prostate-1), which is expressed in most prostate cancers and Ewing sarcomas, as well as in subgroups of other cancers [12]. STEAP1 is associated with tumor invasiveness and progression, which makes it an attractive target for circumventing tumor escape. Recent studies showed that this STEAP1 CAR exhibits potent anti-cancer activity in vitro and in animal models [13, 14]. Thus, following a transient exposure to febrile temperature, the STEAP1 CAR-T cells were co-cultured with cancer cells expressing the STEAP1 antigen. We observed that, like in mice, TPT led to increased production of IFN-γ, an important moderator of T cell mediated cytotoxicity [15], in the human CAR-T cells. However, TPT had no impact on their cell-killing ability, arguing against a broad applicability of TPT for CAR-T therapy.

## Methods

### Cell lines, primary T cell cultures

22Rv1 prostate cancer cell lines were gifts from Prof. Fahri Saatcioglu. The 22Rv1 cells were cultured in RPMI-1640 (Sigma-Aldrich, Oslo, Norway) with 10% heat-inactivated fetal bovine serum (FBS) (Sigma-Aldrich, Oslo, Norway) and 100 U/mL penicillin/streptomycin (Sigma-Aldrich, Oslo, Norway) (complete medium). PBMCs were isolated from healthy donor buffy coats using Lymphoprep (Axis-Shield, Oslo, Norway) and cultured in complete medium.

### T cell activation and heating protocol

STEAP1 CAR T cells were produced by retroviral transduction of PBMCs followed by expansion at 37 °C for 6–7 days, as previously described [13], and frozen at liquid nitrogen. CAR T cells were then thawed and activated using plates coated with 1 mg/mL anti-CD3 (clone OKT3, BioLegend, Oslo, Norway) and 1 mg/mL anti-CD28 (clone CD28.6, eBioscience, Oslo, Norway), in addition to 100 IU/mL rhIL-2 (R&D Systems, Abingdon, UK). Transduced and non-transduced primary T cells were activated either at 37 ^o^C or 39.5 ^o^C for 24 h inn a fully humidified incubator with 5% CO_2_.

### Flow cytometry T cell assays

The CAR and NT-T cells were then frozen after expansion and thawed before use in functional T cell assays. The T cells were activated at 37 ^o^C or 39.5 ^o^C temperatures. For flow cytometry cytokine assays, the T cells were co-cultured with target cells (22RV1) at an E: T ratio of 1:1 for 16 h in the presence of BD GolgiStop and BD GolgiPlug (BD Biosciences, Franklin Lakes, NJ, USA). IFN-γ production in the total CD3 + T cell population was determined by flow cytometry. For flow cytometry killing assays, the T cells were co-cultured with target cell (22RV1) at an E: T ratio of 1:1 for 16 h. The killing efficacy of the CAR-Ts are determined by measuring the caspase 3 production in the target cells using flow cytometry.

### Real-time killing assay

The real-time killing assay was performed with IncuCyte S3 live cell image system (Sartorius AG, Goettingen, Germany). The target cells 22Rv1 expressing GFP were seeded at 1 × 10^5^/well in 96-well plates and irradiated at 20 Gy. The next day, cryopreserved T cells were thawed and added to the culture at E: T ratio of 1:1 and 1:3. The plate was real-time monitored and imaged by IncuCyte S3 every 3 h for 7 days.

### RT-qPCR

RNA was extracted using TRI Reagent Solution. The cDNA synthesis was performed with SuperScript IV Reverse Transcriptase. The qPCR was run on the Biorad Real-Time PCR System using Fast SYBR Green Master Mix using the following primers:

IFN-g Fw: 5’ TGT AGC GGA TAA TGG AAC TCT TTT 3’.

IFN-g Rv: 5’ AAT TTG GCT CTG CAT TAT T 3’.

TNF-a Fw: 5’ CCG AGG CAG TCA GAT CAT CTT 3’.

TNF-a Rv: 5’ AGC TGC CCC TCA GCT TGA 3’.

BATF Fw: 5’ GACAGAGGCAGACACAGAAG 3’.

BATF Rv: 5’ TGCTTGATCTCCTTGCGTAG 3’.

B-actin Fw: 5’ TAA TGT CAC GCA CGA TTT CCC 3’.

B-actin Rv: 5’ TCA CCG AGC GCG GCT 3’.

## Results and discussion

**Fig. 1:**
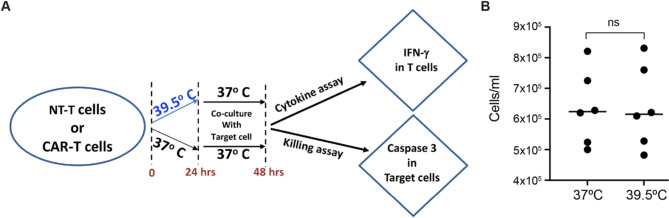
Protocol used to assess the effect of febrile temperature on human T cells. **(A)** The non-transduced T cells (NT-T) or CAR-T cells were activated for 24 h with antibodies against CD3 and CD28 at either 37 ^o^C or 39.5 ^o^C. Next, the T cells were co-cultured with STEAP1 + target cancer cells (22RV1) for 24 h at 37 ^o^C. Then, IFN-γ and caspase 3 positive cells were detected by FACS. **(B)** The numbers of CAR-T cells were determined (by trypan blue staining) after the activation for 24 h at 37 ^o^C or 39.5 ^o^C. Note that temperature had no impact on cell numbers, implying no change in cell proliferation

A previous study has shown that a transient exposure to febrile temperature (39.5 ^o^C for 6 h) can enhance tumor antigen-specific effector functions of murine CD8^+^ T cells, namely the production of IFN-γ and cytotoxicity [16]. To test it in human cells, we examined human T cells from eight healthy donors, expressing a CAR binding the STEAP1 antigen. The STEAP1 CAR construct incorporates a 4-1BB co-stimulatory domain, as previously described [13]. To evaluate the effect of TPT, based on the temperature and time parameters used in previous studies [6, 16], we activated the STEAP1 CAR-T cells with CD3 and CD28 antibodies at either 37 ºC or 39.5 ^o^C for 24 h. Then, the cells were returned to 37 ^o^C and co-cultured with target cancer cells expressing STEAP1 for 24 h (Fig. 1A). We observed no change in cell numbers between the two temperatures (Fig. 1B). The non-transduced T cells from the same donors were used as a negative control and were incubated at 37 ^o^C throughout the experiment.

**Fig. 2:**
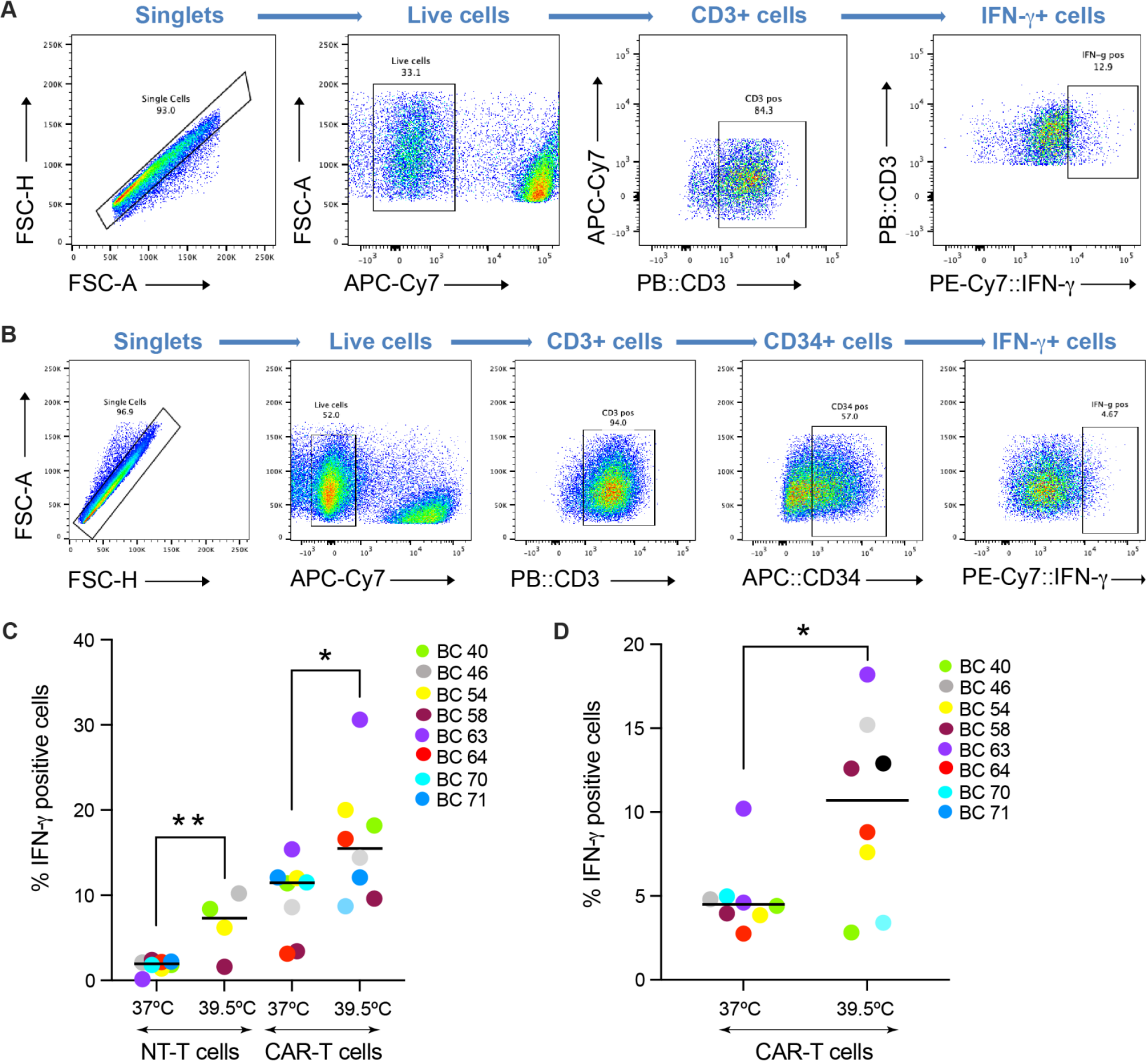
Exposure to febrile temperature potentiates the production of IFN-γ. **(A)** Representative images illustrating a FACS workflow for the detection of IFN-γ positive T cells. Cells derived from donors’ buffy coats (BC) were activated at 37 ^o^C or 39.5 ^o^C and then co-cultured with STEAP1 antigen-expressing target cells. See C for the corresponding quantification. **(B)** Representative images illustrating a FACS workflow for the detection of IFN-γ positive CAR-T cells. Expression of the CAR was determined by staining for the marker protein RQR8, which comprises a CD34 epitope. Cells derived from donors’ buffy coats were activated at 37 ^o^C or 39.5 ^o^C and then co-cultured with STEAP1 antigen-expressing target cells. See D for the corresponding quantification. **(C)** The quantification of A, showing the percentage of IFN-γ positive non-transduced (NT-T) control T cells and CAR-T cells at the indicated temperatures. Colors indicate individual donors. Note that both NT-T cells and CAR-T cells exposed to 39.5 ºC had the tendency to overproduce IFN-γ. **(D)** The quantification of B, showing the percentage of IFN-γ positive CAR-T cells only. Colors indicate individual donors

**Fig. 3:**
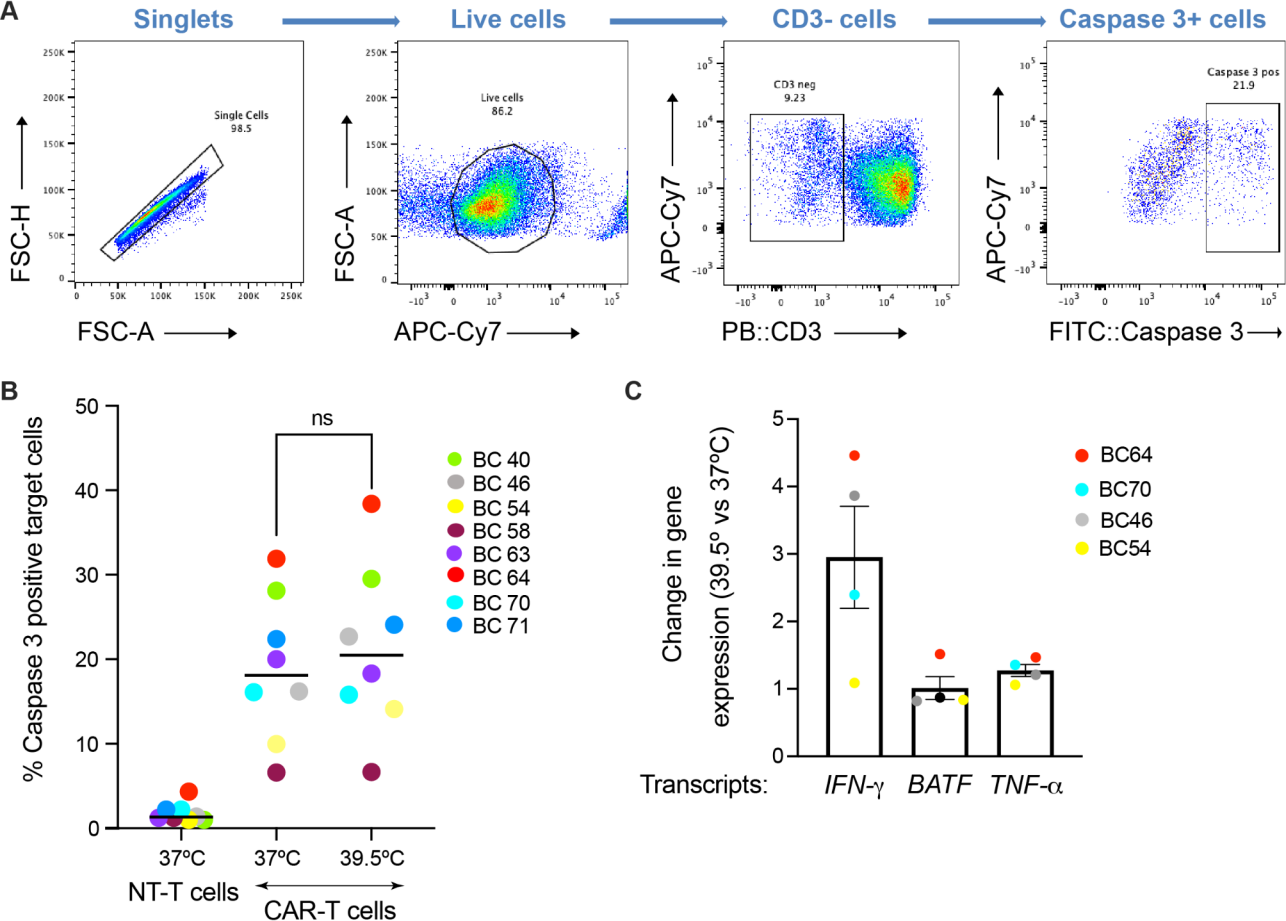
Exposure to febrile temperature does not improve the cell killing ability of STEAP1 CAR-T cells. **(A)** Representative images illustrating a FACS workflow for the detection of caspase 3 positive 22RV1 target cancer cells. The T cells, selected and activated as above, were co-cultured with 22RV1 target cancer cells, after which the induction of caspase 3 in target cells was evaluated. **(B)** The corresponding quantification of caspase 3 positive target cells. Statistical analysis was performed using unpaired T test. Colors indicate individual donors as in A. **(C)** Change in the levels of indicated transcripts in human T cells, detected by RT-qPCR. The cells were harvested before co-culturing with cancer cells (see Fig. 1A)

First, we used flow cytometry to examine the expression of IFN-γ. Although we observed donor-specific differences, TPT tended to increase its production both in the non-transduced T cells and the CAR-T cells (Fig. 2A-D). Thus, a transient exposure to febrile temperature enhances IFN-γ production in both murine and human CD8^+^ T cells. Then, to examine the effect of TPT on cytotoxicity, we performed an in-vitro killing assay. Specifically, the STEAP1 CAR-T cells (treated as in Fig. 1A) were co-cultured with cancer cells expressing STEAP1, and the percentage of dying cancer cells was assessed based on the induction of active caspase 3. In contrast to IFN-γ, we observed no obvious effect of TPT on the killing efficacy of STEAP1 CAR-T cells (Fig. 3A-B).

**Fig. 4:**
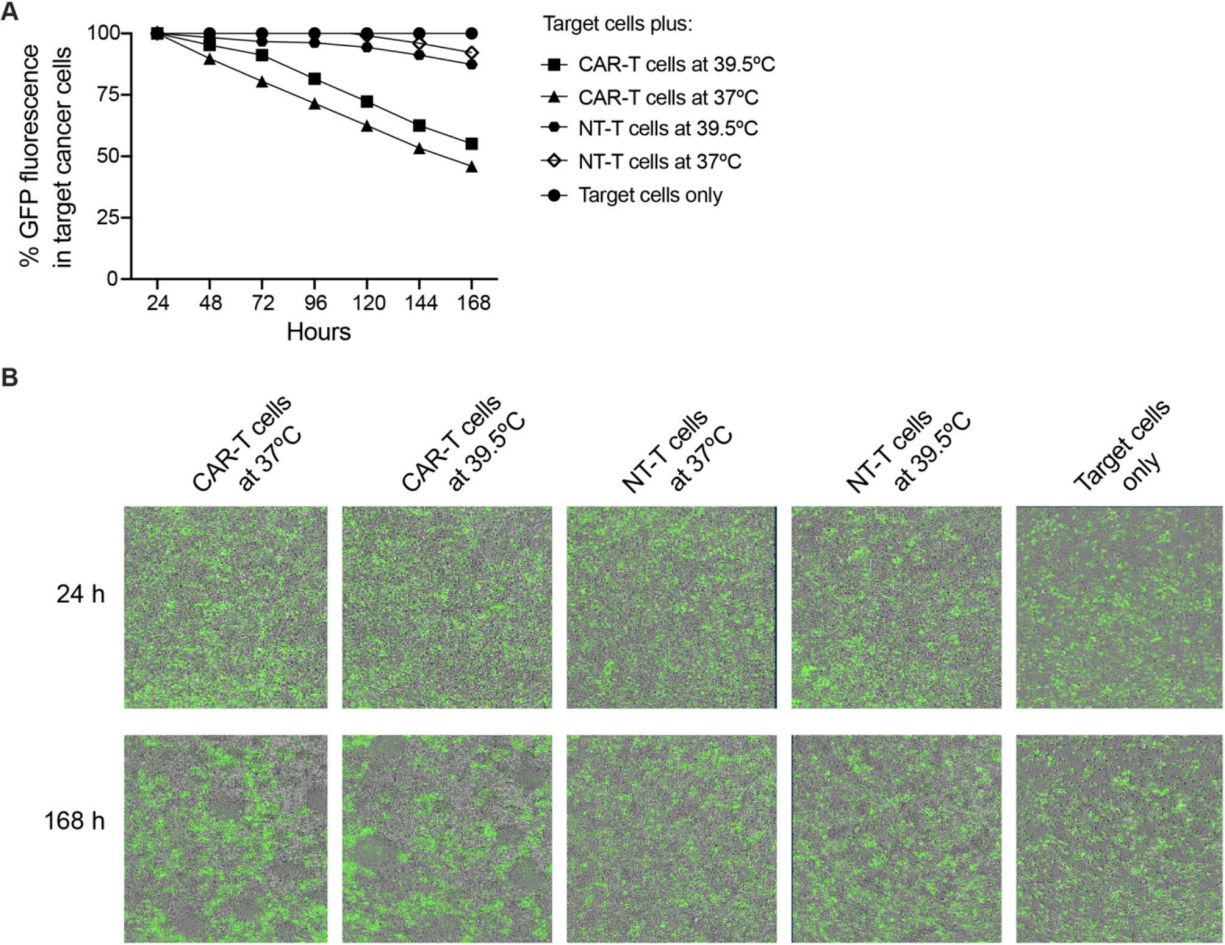
TPT does not improve the killing efficacy of STEAP1 CAR-T cells over a longer time. **(A)** Long-term survival of 22RV1 cancer cells co-cultured with the indicated T cells. The cancer cells were monitored for GFP expression over 7 days by IncuCyte S3. Mean GFP expression was determined by the IncuCyte S3 software and plotted every 24 h. **(B)** Selected IncuCyte images of cells exposed for 24–168 h, as indicated

To corroborate the latter result in another way, we used reverse transcription-quantitative polymerase chain reaction (RT-qPCR) to monitor select transcripts, *TNF-α* and *BATF*, whose expression is associated with antitumor responses. The former encodes TNF-α, a cytokine potentiating the activation and proliferation of the effector T cell [3, 17, 18], and the latter BATF, a transcription factor programming antitumor responses and mitochondrial metabolism [19]. While we observed the expected TPT-dependent upregulation *IFN-γ* mRNA, the levels of *TNF-α* and *BATF* mRNAs did not change (Fig. 3C). In mice, exposure to hyperthermia has long-lasting effects on tumor growth [3]. Thus, we additionally performed a cell-killing experiment over a longer time. We used the IncuSyte S3 real-time live cell analysis, which monitors cell viability by continuously imaging GFP expression. However, also in this assay, we observed no effect of TPT on the killing efficacy of CAR-T cells (Fig. 4A-B).

Summarizing, studies in murine cancer models suggested the possibility that incorporating TPT into existing protocols of CAR-T cell therapy might improve its effectiveness in humans. To test this hypothesis, we used in vitro assays to examine the effect of TPT on the human STEAP1 CAR-T cells. Although we observed TPT-enhanced production of IFN-γ, neither the expression of other tested factors involved in antitumor responses, nor the cell-killing ability of STEAP1 CAR-T cells were improved by TPT. Thus, TPT appears to affect murine and human CD8 + T cells differently. Further investigations will be necessary to understand exactly why. In one scenario, TPT could be incomplete or short-lived in human cells, as we did not continue monitoring IFN-γ levels beyond 48 h. Because plasma membrane plays an important role in temperature-sensing, one could speculate that species-specific outcomes may reflect species-specific changes in the membrane organization and downstream signaling pathways. Irrespective of the explanation, our pilot studies suggest that applying TPT to humans, at least in conjunction with the CAR-T cell therapy, may be more complicated than anticipated.

## Limitations

A different choice of TPT protocol or CAR could impact the outcome of this study.

## Data Availability

All data generated or analyzed during this study are included in this published article.

## Abbreviations

CAR: chimeric antigen receptor
CAR-T: chimeric antigen receptor T-cell
STEAP1: six transmembrane epithelial antigen of prostate-1
TPT: thermal potentiation of T cells
